# Successful vaginal birth after two or more cesareans versus failed trial of labor: A systematic review and meta-analysis of maternal and fetal outcomes

**DOI:** 10.61622/rbgo/2026rbgo48

**Published:** 2026-09-23

**Authors:** Beatriz Nascimento Motta Spaleta, Juliana Almeida Oliveira, Laura Fonseca Queiroz, Aline Torres Alves, Caroline Otoni Durães, Aline Evangelista Santiago, Lucas Ribeiro de Carvalho

**Affiliations:** 1 Universidade Federal de Minas Gerais Department of Surgery Belo Horizonte MG Brazil Department of Surgery, Universidade Federal de Minas Gerais, Belo Horizonte, MG, Brazil.; 2 Universidade Santo Amaro Department of Obstetrics and Gynecology São Paulo SP Brazil Department of Obstetrics and Gynecology, Universidade Santo Amaro, São Paulo, SP, Brazil.; 3 Santa Casa de Misericórdia Department of Obstetrics and Gynecology Belo Horizonte MG Brazil Department of Obstetrics and Gynecology, Santa Casa de Misericórdia, Belo Horizonte, MG, Brazil.; 4 Universidade Estadual de São Paulo Department of Tocogynecology Botucatu SP Brazil Department of Tocogynecology, Universidade Estadual de São Paulo, Botucatu, SP, Brazil; 5 Irmandade Nossa Senhora das Graças de Sete Lagoas Department of Obstetrics and Gynecology MG Brazil Department of Obstetrics and Gynecology, Irmandade Nossa Senhora das Graças de Sete Lagoas, MG, Brazil.; 6 Universidade Federal da Fronteira do Sul Department of Obstetrics and Gynecology Chapecó SC Brazil Department of Obstetrics and Gynecology, Universidade Federal da Fronteira do Sul, Chapecó, SC, Brazil.

**Keywords:** Vaginal birth after cesarean, Cesarean section, repeat, Trial of labor, Uterine rupture, Pregnancy complications, Risk factors, Risk assessment, Protective factors, Pregnant women, Pregnancy outcome

## Abstract

**Objective:**

To assess maternal and neonatal outcomes associated with a failed versus successful (VBAC-2).

**Data sources:**

A systematic search across Pubmed, Embase and Cochrane was conducted up to October 2025, supplemented by backward snowballing.

**Study selection:**

Studies with pregnant women with a two or more previous cesarean deliveries undergoing a trial of labor were included. No restrictions were applied.

**Data collection:**

Data on maternal and neonatal outcomes and risk and protective factors were extracted. The DerSimonian and Laird random effects model was applied, reporting Odds Ratios (OR) with 95% confidence intervals. Heterogeneity was evaluated using I^2^ statistics and the Cochran Q test. Methodological quality was appraised using the Cochrane Risk-of-bias tool for non-randomized trials (ROBINS-I).

**Data synthesis:**

Out of 4065 studies screened, 12 studies (n= 3429) were included. We observed higher odds of postpartum hemorrhage (OR 1.98), maternal transfusion (OR 3.29), uterine rupture (OR 6.47), and neonatal intensive care unit admission (OR 6.44) in the VBAC-2 failure group. Epidural anesthesia (OR 0.56) and a history of previous vaginal birth (OR 0.27) were identified as protective factors, while a higher Body Mass Index was a risk factor for VBAC- 2 failure.

**Conclusions:**

These findings highlight the critical need for comprehensive patient counseling, risk assessment, and heightened clinical vigilance to optimize care strategies in this high-risk population.

## Introduction

Cesarean section rates have been steadily rising globally,^(1)^ being projected that by 2030, 1 in every 3 women will have a cesarean delivery,^(2)^ leading to a concomitant increase in the number of women with a history of multiple previous cesarean deliveries.^(3)^ While vaginal birth after one prior cesarean (VBAC-1) has become a well-established and often recommended option for eligible women with success rates of 60 to 80%,^(4,5)^ the safety and feasibility of vaginal birth after two or more previous cesareans (VBAC-2 or more) remain areas of considerable debate and clinical uncertainty.^(6)^ Current guidelines often provide limited or conflicting recommendations regarding trials of labor (TOL) in this specific high-risk population, primarily due to concerns about potentially increased maternal and neonatal complications, particularly uterine rupture.^(7)^

A successful VBAC-2 attempt offers numerous benefits, including avoidance of repeat abdominal surgery, reduced risks of future placental complications, and a shorter recovery period.^(4)^ However, the potential for a failed trial of labor is a significant consideration, as it often culminates in an emergency repeat cesarean section, which can carry greater risks than a planned repeat cesarean.^(8)^ Understanding the differential outcomes between a successful VBAC-2 and a failed TOL after cesarean (TOLAC) in women with two or more previous cesareans is paramount for accurate patient counseling, informed decision-making, and the development of evidence-based clinical guidelines.

Existing literature often focuses on the overall success rates of VBAC-2 or aggregates outcomes without a direct comparison between the two distinct labor courses.^(9)^ A clear understanding of the risks associated with a failed VBAC-2 attempt, compared to a successful one, is crucial for clinicians and patients to weigh the benefits against the potential morbidities.

This systematic review and meta-analysis aimed to precisely delineate the maternal and neonatal outcomes associated with a failed trial of labor after two or more previous cesareans compared to a successful vaginal birth in pregnant women. The review's objective was developed using the PICO (acronym for Population, Intervention, Control and Outcome) framework, further described in the eligibility criteria.

## Methods

### Study protocol and registration

This systematic review and meta-analysis adhered to the guidelines provided by the Cochrane Collaboration^(10)^ and the Preferred Reporting Items for Systematic Reviews and Meta-Analyses (PRISMA).^(11)^ The review's detailed protocol was formally registered in advance with the International Prospective Register of Systematic Reviews (PROSPERO) in October 2025, under the identification number: CRD420251161382.

### Search strategy

A comprehensive search was conducted across electronic databases including PubMed, Embase, and the Cochrane Central Register of Controlled Trials during October 2025. The search strategy employed the Medical Subject Headings (MeSH) term "vaginal birth after two cesareans" alongside other related vocabulary and is available Chart 1S. To further identify pertinent research, a "backward snowballing" technique was applied, involving the examination of reference lists from previously published works. Non-retrieved work was requested to the authors, with no response until the day of publication.

### Eligibility criteria

Studies were deemed eligible for inclusion if they fulfilled the following criteria: (1) involved a study population exclusively comprising pregnant women; (2) featured a direct comparison between vaginal birth after two or more cesareans success *versus* failure of vaginal birth in the same population; and (3) prospective or retrospective studies. Due to the nature of the study, we did not limit the type of trial. No restrictions were imposed based on publication date or language. Studies were excluded if they involved patient cohorts that overlapped with other included research and if they were conference proceedings, editorials, and letters to the editor. The screening of identified studies was carried out independently by two authors, strictly adhering to the predetermined search and eligibility criteria. Any discrepancies arising between the authors were resolved through thorough discussion and consensus (Chart 1S).

### Data collection

Data pertaining to study outcomes and baseline characteristics were independently extracted by two investigators and checked by a third author. A rigorous cross-verification process was undertaken to ensure the consistency and accuracy of the collected data for subsequent statistical analysis. Patient-level data was not sought for this review. The outcomes of interest were: (1) post-partum hemorrhage; (2) uterine rupture; (3) hysterectomy; (4) maternal transfusion; (5) APGAR below 7; (6) Neonatal Intensive Care Unit (NICU) admission; and (7) neonatal death. We also assessed if any risk or protective factors were more prevalent in one of the groups, such as: (1) previous vaginal birth; (2) number of pregnancies with birth (parity); (3) gestational age; (4) maternal age at birth; (5) body mass index (BMI); (6) personal history of diabetes (type I, type II or gestational diabetes - GDM); (7) epidural anesthesia; (8) time since the last cesarean; and (9) neonatal weight at birth. Due to the low amount of data, no subgroup analyses were performed. The definition of all outcomes was shared in the chart 2S, for clarity. The outcomes of hysterectomy, neonatal death, and an Apgar score below 7 were not explicitly defined in the included studies and were not shared in Chart 2S. This may reflect their inherently objective nature, as uterine removal, mortality, and Apgar scores are less susceptible to subjective interpretation or measurement.

### Data synthesis

The DerSimonian and Laird random effects model was employed for the synthesis of binary outcomes. Odds ratios (OR) accompanied by their 95% confidence intervals (CI) served as the chosen measures of effect size. Adjusted odds ratio was not performed. Heterogeneity among studies was evaluated using the I^2^ statistic and the Cochran Q test; heterogeneity was considered statistically significant if the p-value was less than 0.10 and the I^2^ value surpassed 25%. Sensitivity analyses were performed through a leave-one-out approach and the generation of Baujat plots. Statistical computations were executed using Review Manager 5.1 (Nordic Cochrane Centre, The Cochrane Collaboration, Copenhagen, Denmark) and RStudio (PBC, Boston, MA). Meta-regression and subgroup analysis were inhibited due to the lack of data, since at least 10 studies with at least one event are recommended and studies did not share enough data enabling those analyses. Importantly, the study's design was limited by the research question, which inhibits randomized designs. The methodological quality of the included studies was appraised using the Cochrane Risk-of-bias tool for non- randomized trials (ROBINS-I).^(13)^ This risk assessment was conducted independently by two authors (JAO and AT), with any discrepancies resolved through detailed discussion. Given the limited number of studies identified and according to Cochrane recommendations, it was not feasible to conduct an assessment for small study effects (publication bias). The plots were generated through the Risk of Bias visualization tool (Robvis).^(14)^

## Results

### Study selection

After screening a total of 4065 studies (Figure 1), 12 studies were included in this meta- analysis, comprising 3429 women, of whom 887 (25.9%) failed vaginal birth after two or more cesareans.^(12,15-21,21-25)^ Only one study had a prospective design^(17)^ and most of them were conducted in Europe.^(15,18,20,21,23,24)^ Importantly, only one study included patients with two or more cesareans.^(12)^ All others included those with two previous cesarean births.

**Figure 1:**
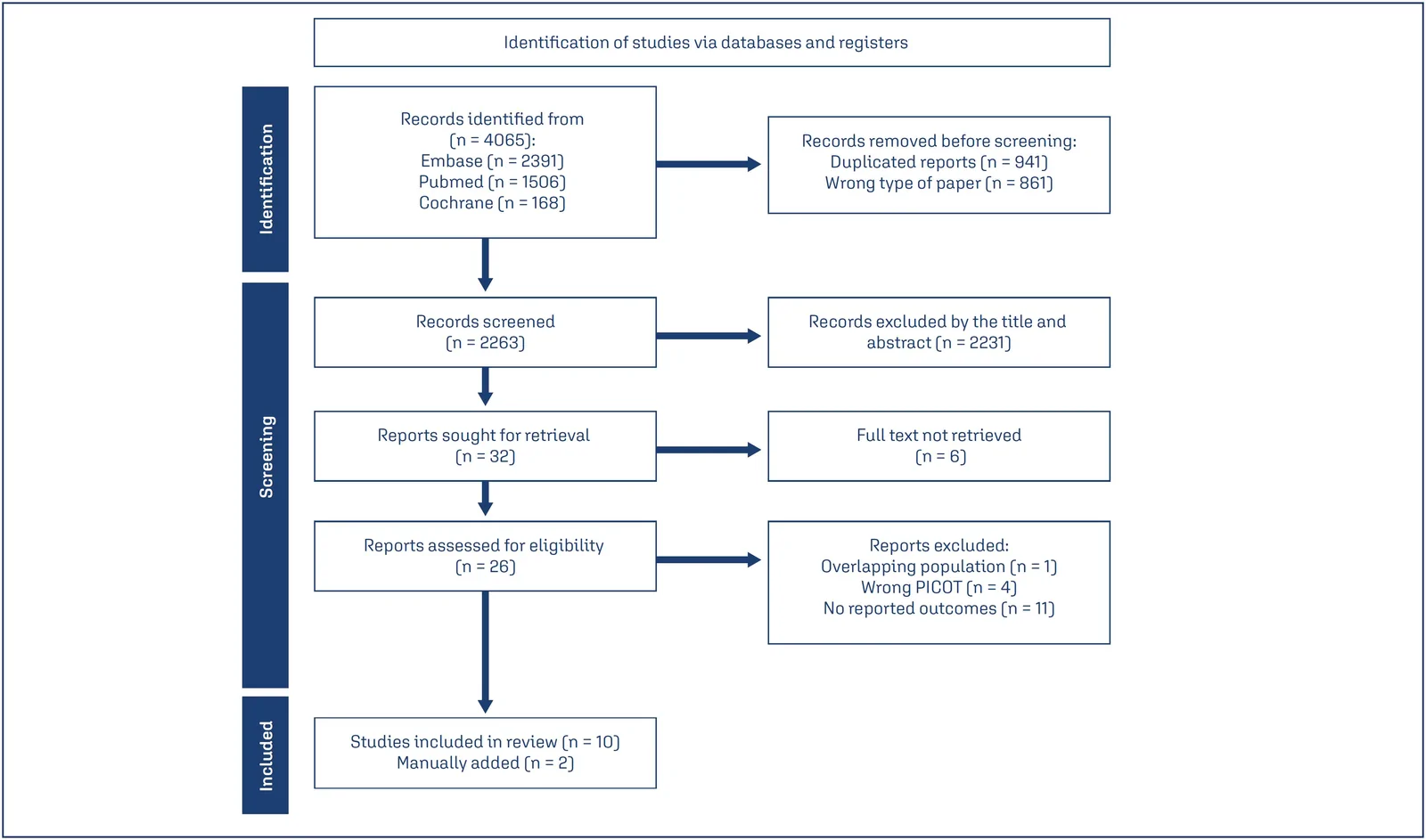
Screening process

### Study characteristics

The study's main characteristics can be seen in chart 2.

**Chart 2:** Included studies main characteristics

	Country	Type of study	N. of patients, Failure / Success, N (%)	Maternal Age, Failure / Success,^a^ years	Previous history of vaginal birth, n(%)	GA at birth, Failure / Success,^a^ weeks	Weight at birth, Failure / Success,^a^ grams
Asakura 1995^(12)^	USA	Retrospective observational	109(35.7%) / 196(64.3%)	NA	8(7%) / 59(30%)	37 / 38^b^	2080 / 2996^b^
Bretelle 2001^(23)^	France	Retrospective cohort	33(34.4%) / 63(65.6%)	NA	NA	NA	NA
De Leo 2020^(21)^	Italy	Retrospective observational	11(24%) / 35(76%)	35.9 ± 3.81 / 34.3 ± 5.58	1(9%) / 13(37%)	NA / 40.34 ± 1.53	3741 ± 458 / 38.94 ± 1.64
Denjean 2024^(24)^	Germany	Retrospective cohort	41(45%) / 50(55%)	34 (25 to 44) / 35 (21 to 44)	5(12%) / 12(24%)	40.17 ± 1.25 / 39.68 ± 1.20	3680 (2680 to 4650) / 3535 (2680 to 4480)
Horgan 2022^(22)^	USA	Retrospective case -control	25(30.5%) / 57(69.5%)	32.8 ± 5.1 / 33.2 ± 4.6	7(28%) / 43(75.4%)	NA	3681 ± 445.1 / 3350.9 ± 362.1
Maroyi 2023^(17)^	Congo	Prospective cohort	127(34.3%) / 405(65.7%)	30 ± 7	NA	39b	NA
Metz 2015^(16)^	USA	Retrospective observational	126(34.1%) / 243(65.9%)	29.6 ± 5.3 / 29.5 ± 5.7	33(26.2%) / 154(63.4%)	39.6 ± 1.43 / 39.4 ± 1.29	3462 ± 52 / 3291 ± 29.75
Modzelews 2019^(15)^	Poland	Retrospective observational	13(37.1%) / 22(62.9%)	33.85 ± 3.26 / 32.45 ± 4.27	NA	40 ± 1.08 / 39.91 ± 0.92	3886.15 ± 448.75/ 3760.68 ± 392.50
Rotem 2021^(19)^	Israel	Retrospective cohort	67(13.8%) / 418(86.2%)	34.82 ± 4.8 / 35.02 ± 3.24	NA	NA	NA
Roux 2023^(18)^	France	Retrospective cohort	17(32.7%) / 35(67.3%)	33.29 ± 2.24 / 33.5 ± 3.13	11(64.7%) / 28(80%)	NA	50.8 ± 28.6 / 51.9 ± 25.4c
Spaans 2003^(20)^	Netherlands	Retrospective cohort	10(18.2%) / 45(81.8%)	NA	NA	NA	NA
Gold Zamir 2024^(25)^	Ireland, UK	Retrospective cohort	208(26.1%) / 973(73.9%)	34 ± 4.8 / 34.5 ± 4.6	132(63.5%) / 886(91%)	38.3 ± 2.1 / 39.5 ± 1.3	3190.9 ± 585.6 / 3406.9 ± 467.2

a Mean and standard deviation

b No standard deviation available

c The data was shared in percentile; UK: United Kingdom; USA: United States of America; NA: Not available

### Pooled analysis of results comparing VBAC-2 failure with success

Our pooled analysis showed that women who failed vaginal birth after two or more cesareans presented significant higher odds of hemorrhage (OR 1.98, 95% CI 1.24 to 3. 16, p= 0.0004; I2 = 0%) (Figure 2), need of transfusion (OR 3.29, 95% CI 1.38 to 7.86, p= 0.007; I2 = 0%) (Figure 3), and uterine rupture (OR 6.47, 95% CI 2.36 to 17.74, p < 0.001; I2 = 0%) (Figure 4). As for fetal outcomes, we observed higher rates of neonatal ICU admission in the VBAC-2 failure group (OR 6 .44, 95% CI 2.72 to 15.29, p < 0.001; I2 = 60.8%; Figure 5). Other outcomes were assessed with no significant findings, such as APGAR below 7 (OR 3.55, 95% CI 0.79 to 15.96; I2 = NA (Supplemental material figure 1S) and neonatal death (OR 0.2, 95% CI 0.01 to 4. 19, p= 0.3; I2 = NA) (Supplemental material figure 2S).

**Figure 2:**
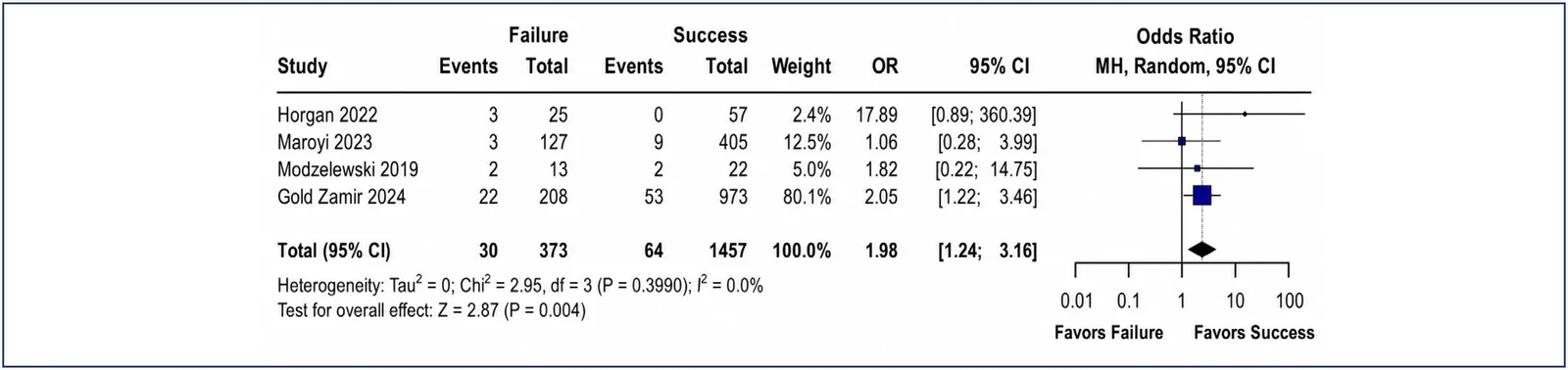
Patients who failed VBAC-2 presented higher odds of hemorrhage when compared to those who succeeded in vaginal delivery

**Figure 3:**
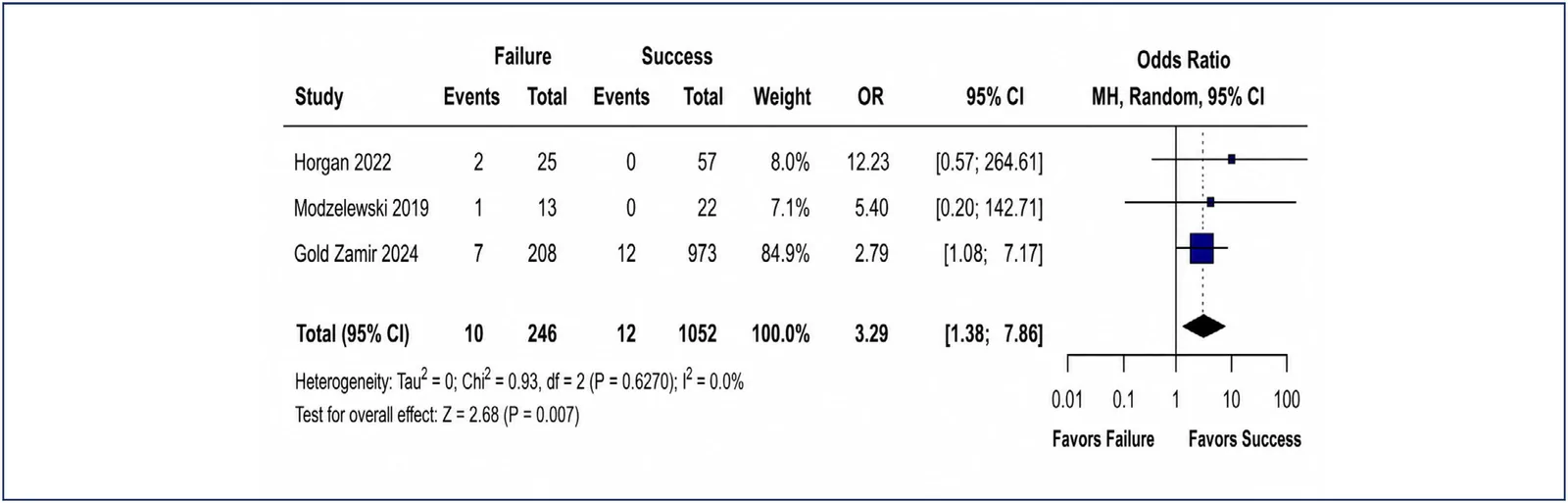
Transfusion was three times more frequent in women who were not able of having a vaginal birth after two or more cesareans when compared to those who succeeded

**Figure 4:**
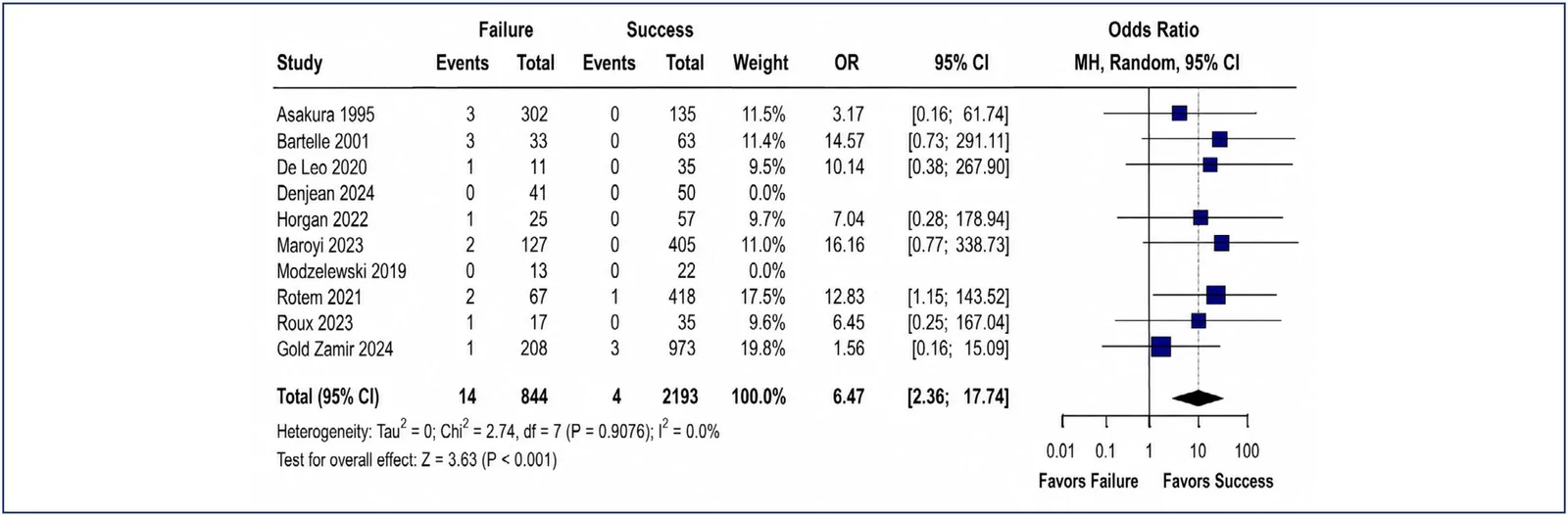
Patients who failed VBAC-2 had higher odds of uterine rupture when compared to those who succeeded in vaginal delivery

**Figure 5:**
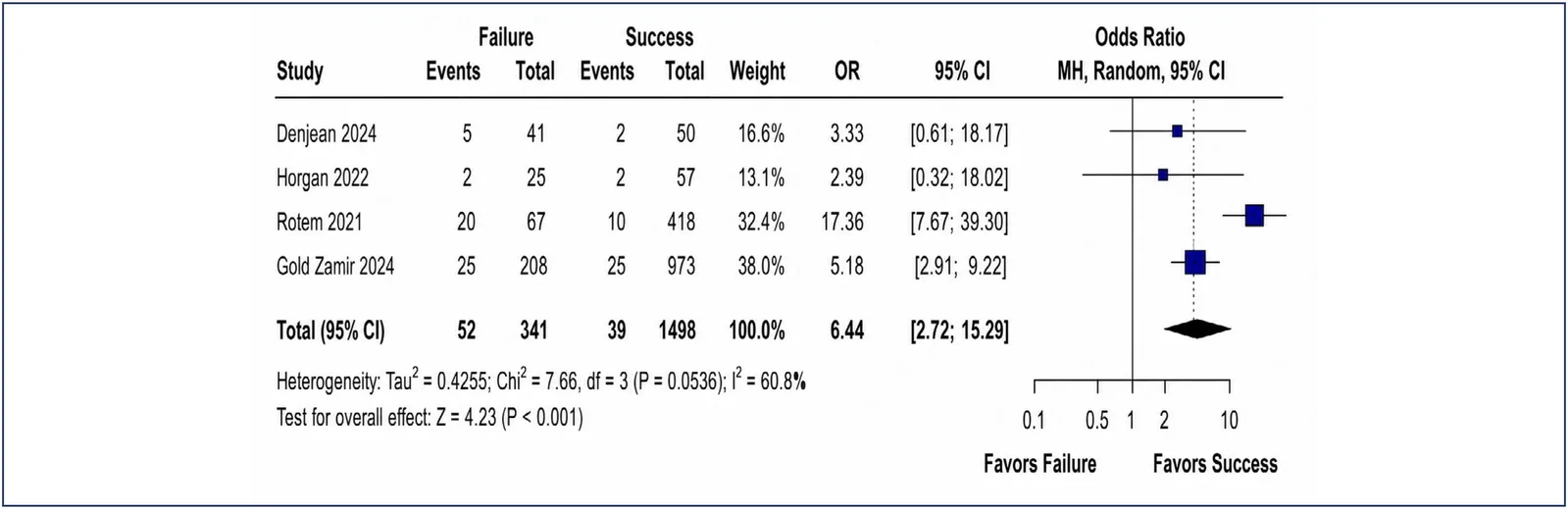
Offspring of mothers who failed vaginal birth had six times higher odds of neonatal ICU admission compared to those who succeeded

### Risk factors assessment

We also observed the common protective and risk factor associated with failure or success of vaginal birth after two or more cesareans. We observed that epidural anesthesia decreased the odds of VBAC-2 failure (OR 0.56, 95% CI 0.39 to 0.80, p = 0.001; I2 = 31.9%) (Figure 6) and that previous vaginal birth was a protective factor (OR 0.27, 95% CI 0.16 to 0. 45, p < 0.001; I2 = 65.8%) (Figure 7). Higher BMI was associated with VBAC-2 failure (OR 1.51, 95% CI 0.24 to 2.79, p= 0.02; I2 = 61.8%) (Figure 8). Other non-statistically significant potential risk factors assessed were child weight at birth (OR 60.35, 95% CI -145.90 to 266.60, p = 0.57; I2 = 89%) (Supplemental Material figure 3S), time since the last cesarean (OR -0.05, 95 % CI -0.98 to 0.88, p= 0.92; I2 = 97.2%); (Supplemental Material figure 4S), gestational age at delivery (OR -0. 13, 95% CI -0.86 to 0.61, p= 0.74; I2 = 92.6%); (Supplemental Material figure 5S), mean parity (OR -0.73, 95% CI -1.48 to 0.02, p= 0.06; I2 = 80.2%) (Supplemental Material figure 6S) and diabetes, despite studies with various types (I, II and gestational diabetes) which impacted on heterogeneity (OR 1.57, 95% CI 0.61 to 4.01, p= 0.349; I2 = 70.4%) (Supplemental Material figure 7S).

**Figure 6:**
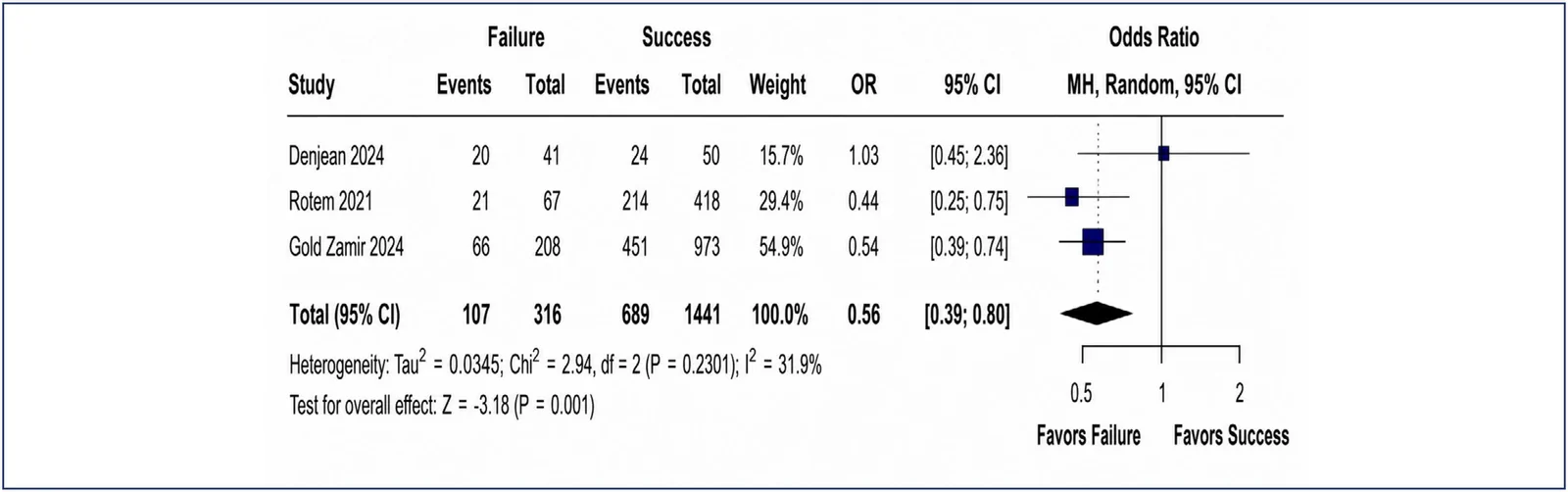
Patients who failed VBAC-2 had lower odds of being exposed to epidural anesthesia when compared to those who achieved vaginal birth

**Figure 7:**
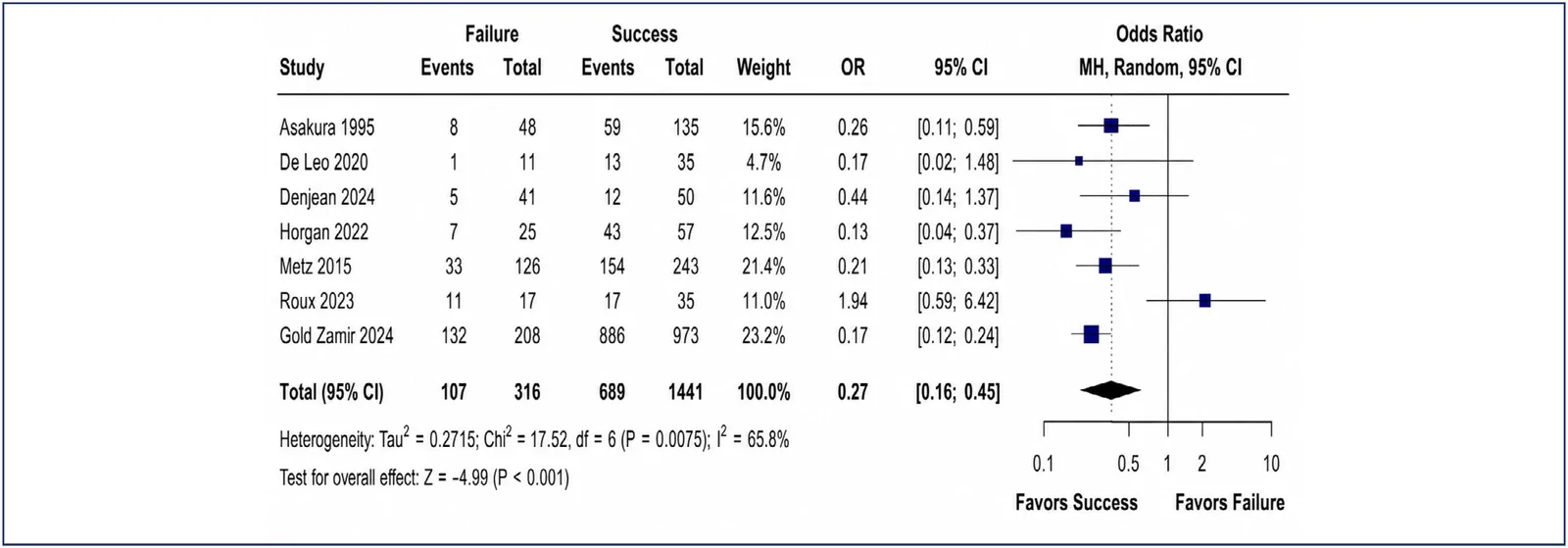
Patients who succeeded VBAC-2 had higher odds of having a history of previous vaginal birth when compared to those who failed

**Figure 8:**
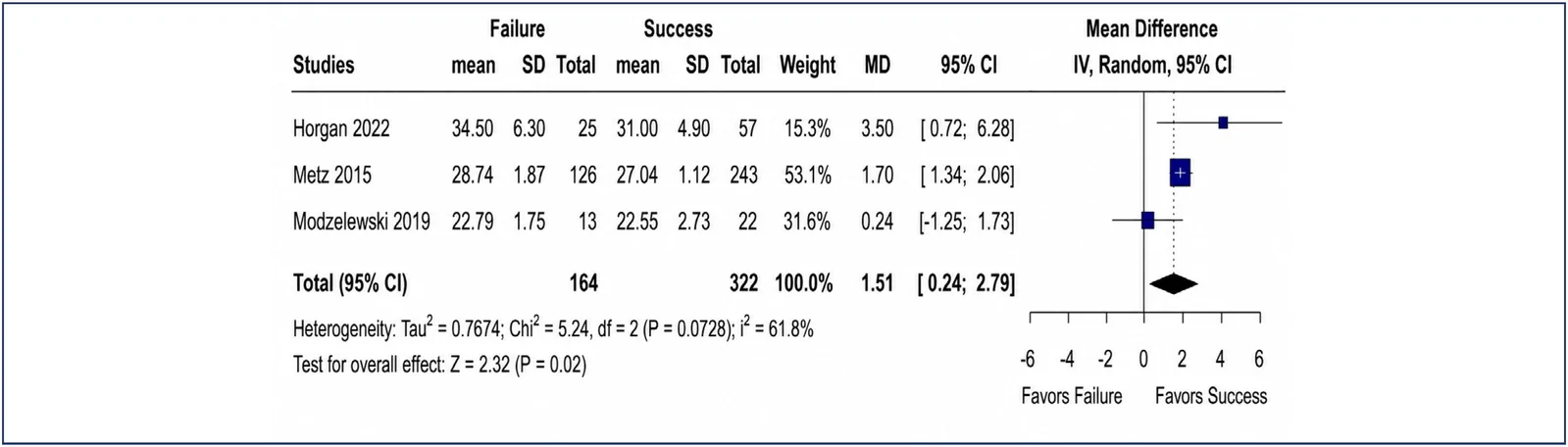
Higher body mass index was associated with VBAC-2 failure when compared to women who succeeded to achieve vaginal delivery

### Sensitivity analysis and quality assessment

Our sensitivity analysis showed that one study was associated with substantial heterogeneity for the outcome of neonatal ICU admission^(19)^(Supplemental Material figure 8S), and another for the risk factors of diabetes, gestational age, time since the last cesarean section,^(25)^ previous vaginal delivery,^(18)^ and peridural anesthesia^(24)^(Supplemental Material figure 9S-13S). All the other assessments did not assign to increased heterogeneity (Supplemental Material figure 14S-22S).

Eleven out of the 12 included studies were classified with overall serious risk of bias, and one with moderate risk.^(16)^ Most studies presented bias due to residual confounding by unmeasured or inadequately adjusted variables. Due to the nature of the study, bias in selection of participants was serious, mostly due to choice of treatment driven by patient preference, physician counseling, and hospital guidelines. We do understand the importance of such, but it leads to a high selected study population, and to an inherent methodological challenge. Other domains remained with low risk of bias (Figure 9). Traffic and summary plots for quality assessment can be seen in the figure 10.

**Figure 9:**
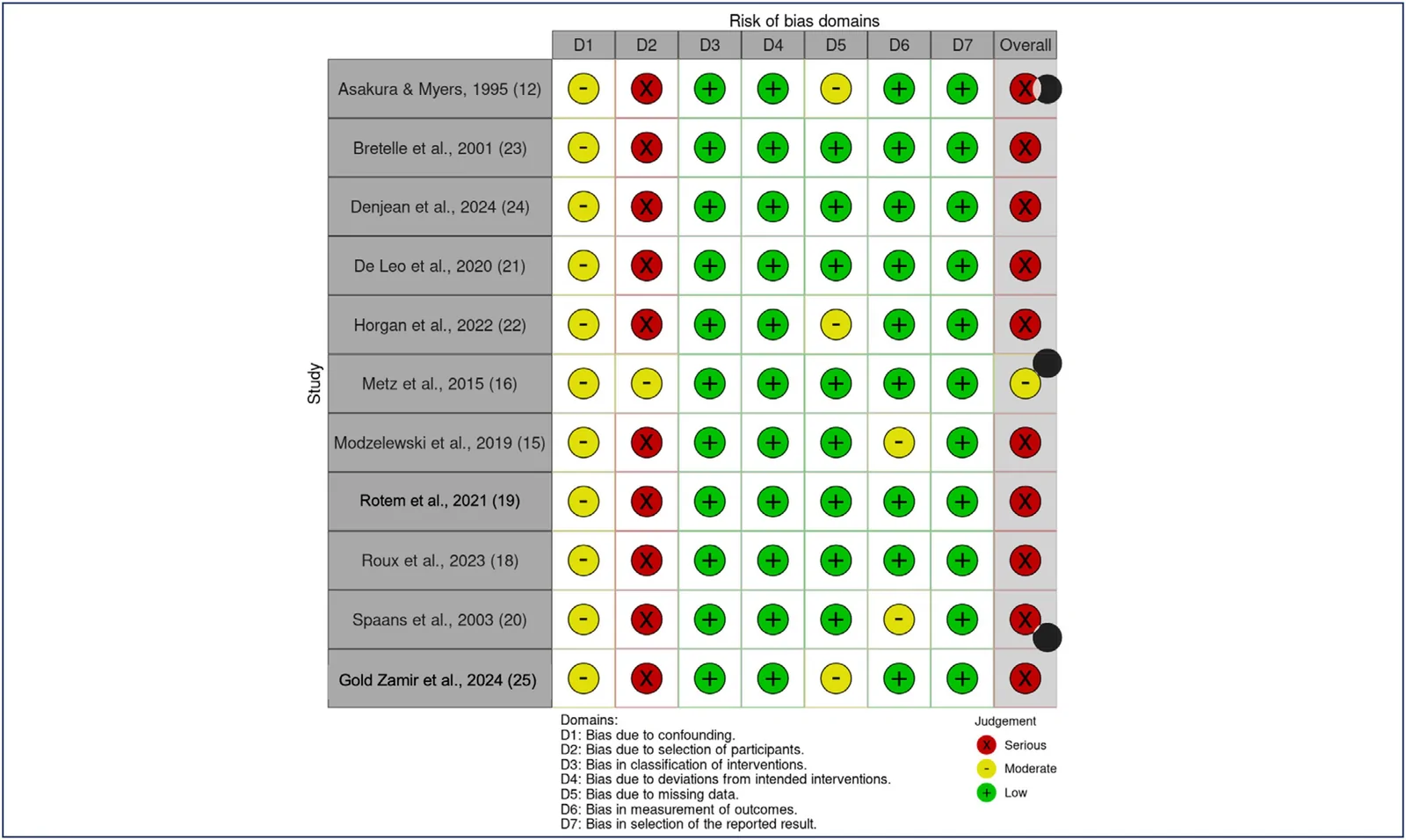
Traffic-light plot for quality assessment

**Figure 10:**
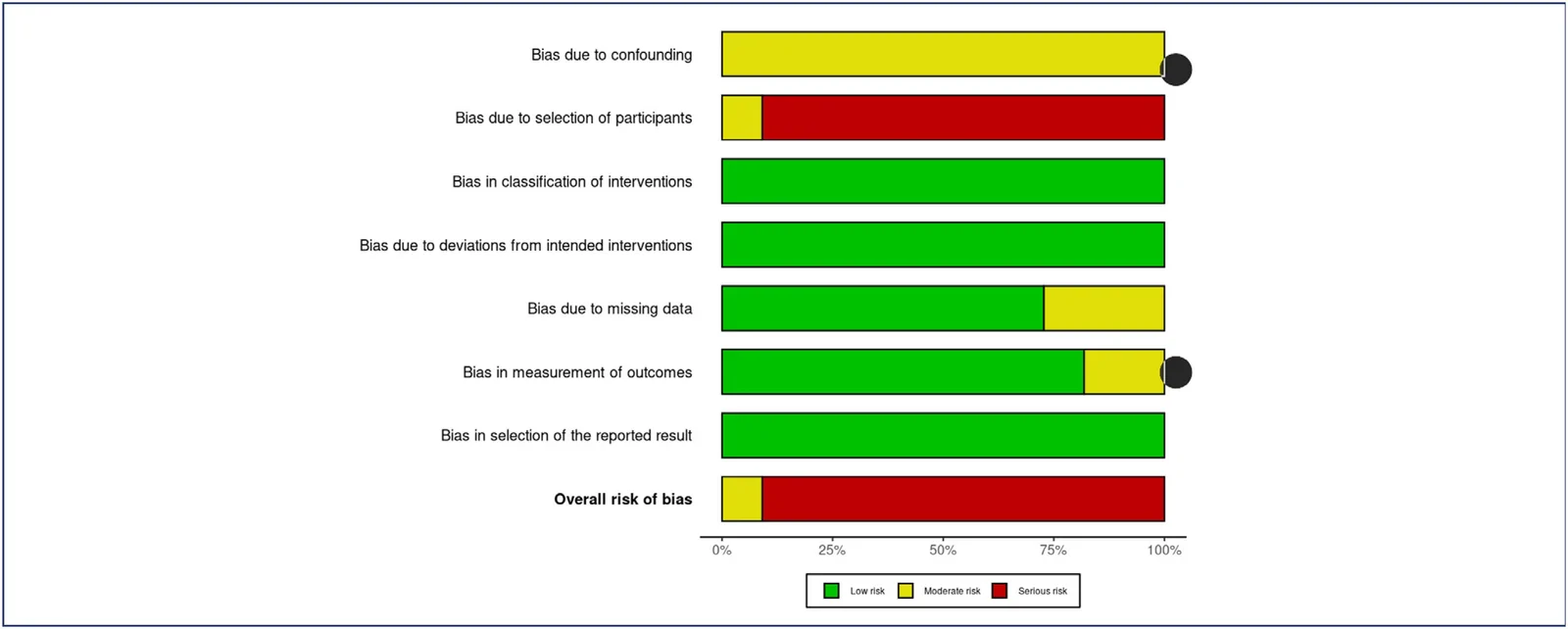
Summary plot for quality assessment

## Discussion

This systematic review and meta-analysis provides crucial insights into the maternal and neonatal outcomes following a failed trial of vaginal birth after two or more previous cesarean sections (VBAC-2) compared to successful VBAC-2. Our pooled analysis revealed statistically significant and clinically important differences in several key outcomes. Women experiencing a failed VBAC-2 attempt faced nearly twice the odds of postpartum hemorrhage (OR 1. 98) and over three times the odds of requiring a maternal transfusion (OR 3.29) compared to those with a successful VBAC-2. Most strikingly, the odds of uterine rupture were more than six times higher in the failure group (OR 6.47). Regarding neonatal outcomes, NICU admission rates were also significantly elevated, with more than a six-fold increase in odds following a failed VBAC-2 (OR 6.44). Other outcomes did not show statistically significant differences, potentially due to lower incidence or limited data.

The elevated risks of postpartum hemorrhage and maternal transfusion in cases of failed VBAC-2 are critical findings with direct implications for clinical practice. Postpartum hemorrhage remains a leading cause of maternal morbidity and mortality worldwide,^(26,27)^ often necessitating more innovative approaches^(28)^ and intensive medical intervention, including blood product transfusion, and in severe cases, hysterectomy.^(29,30)^

Understanding that a failed VBAC-2 attempt significantly increases these odds allows clinicians to better counsel patients, optimize resource allocation, and prepare for potential complications.^(28,31)^ Proactive management strategies, such as ensuring availability of blood products and rapid response teams, become even more paramount when caring for women attempting VBAC-2, given the baseline risks associated with the procedure.

Uterine rupture is perhaps the most dreaded complication of VBAC, carrying severe risks of maternal and fetal morbidity and mortality,^(32)^ despite long-term studies not showing clear correlations in cases of one previous cesarean.^(33)^ Our finding of a more than six-fold increase in the odds of uterine rupture in the VBAC-2 failure group (OR 6.47, 95% CI 2.36 to 17.74) highlights the catastrophic potential of this outcome. While still a relatively rare event, its association with failed VBAC-2 attempts demands meticulous patient selection, continuous monitoring during labor, and immediate access to emergency surgical facilities. This finding reinforces the need for robust guidelines and shared decision-making processes that fully inform patients about this specific, life-threatening risk.

The significantly higher odds of neonatal ICU admission (OR 6.44) for offspring of mothers who experienced failed VBAC-2 indicates a substantial impact on neonatal health. NICU admissions can be a proxy for various neonatal complications, including birth asphyxia, respiratory distress, and sepsis, all of which can lead to short-term and long- term developmental challenges.^(34)^ This finding suggests that failed VBAC-2 attempts are not only associated with maternal complications but also contribute to increased neonatal vulnerability and resource utilization. These outcomes necessitate careful consideration during antenatal counseling and heightened vigilance in the immediate postpartum period for infants whose mothers have undergone a failed VBAC-2.

Our analysis of risk factors revealed intriguing associations. We observed that epidural anesthesia decreased the odds of VBAC-2 failure (OR 0.56), suggesting it acts as a protective factor, a finding previously reported in the literature.^(35)^ While potentially reducing contraction intensity and frequency in the second stage of labor, it also can slow down uterine blood flow reducing bleeding and, considering a scarred uterus reducing uterine activity might diminish the risk of uterine rupture,^(36)^ but these findings are still controversial.^(37)^ Furthermore, a history of previous vaginal birth was confirmed as a protective factor (OR 0.27) against VBAC-2 failure, while higher BMI was associated with VBAC-2 failure (OR 1.51).

Despite its comprehensive nature, this meta-analysis is subject to several limitations. A significant limitation stems from the predominantly retrospective design of the included studies, with only one prospective study identified. Retrospective studies are inherently prone to various biases, particularly selection bias and confounding by unmeasured or inadequately adjusted variables. The methodological quality assessment using the ROBINS-I confirmed this, with eleven out of twelve included studies classified with an overall serious risk of bias. Additionally, our sensitivity analysis showed small heterogeneity indicating risk factors variability that could influence findings. Despite being the largest pool of patients, meta-regression and subgroup analysis were inhibited due to the lack of data. The study's design was also limited by the research question, which inhibits randomized designs. However, prospective analysis asks for more detailed collection of data and pre-defined criteria, which enhances the studies quality and level of confidence.

This systematic review and meta-analysis boasts several notable strengths. It adhered rigorously to the guidelines provided by the Cochrane Collaboration and PRISMA, ensuring a robust methodological framework. The detailed protocol prospectively registered with PROSPERO enhances transparency and reduces the risk of reporting bias. A comprehensive search strategy was employed across major electronic databases including PubMed, Embase, and the Cochrane Central Register of Controlled Trials, supplemented by a "backward snowballing" technique, to identify all pertinent literature. Importantly, the review imposed no restrictions based on publication date or language, ensuring a broad inclusion of available evidence. These methodological strengths contribute to the reliability and generalizability of our findings within the confines of the available literature.

Given the significant findings and identified limitations, future research should prioritize robust, prospective study designs, ideally multicenter cohort studies, to minimize selection bias and better control for confounding variables inherent in retrospective analyses. Particular attention should be paid to the observed associations with epidural anesthesia and a history of previous vaginal birth; future studies should aim to thoroughly investigate potential confounding factors, explore the underlying physiological mechanisms, or consider if these associations are artifacts of unmeasured biases. Additionally, studies powered sufficiently to allow for granular subgroup analyses are needed to identify specific patient characteristics that might predict success or failure in VBAC-2, and to better understand outcomes with lower incidence like neonatal death and APGAR scores. Standardized reporting of baseline characteristics, labor management protocols, and comprehensive maternal and neonatal outcomes would greatly enhance the quality and comparability of future meta-analyses.

## Conclusion

This systematic review and meta-analysis indicate that a failed trial of labor after two or more previous cesarean sections (TOLAC-2) might be associated with an increased risk profile for both maternal and neonatal morbidity. Our pooled estimates suggest that women experiencing a failed VBAC-2 attempt may face significantly higher odds of severe complications, such as postpartum hemorrhage, maternal transfusion, and uterine rupture, while neonates appear to be at an elevated risk for NICU admission. However, the certainty of evidence is low, primarily limited by the retrospective nature of the constituent studies and a serious risk of bias identified in the majority of the included literature. While factors such as epidural anesthesia and a history of previous vaginal birth emerged as potential protective elements, and elevated BMI as a risk factor, these associations remain hypothesis-generating due to residual confounding and confounding by indication. Consequently, these findings should not be interpreted as a contraindication to TOLAC-2, but rather as a mandate for individualized risk stratification, robust shared decision-making, and clinical vigilance. Further prospective, multi-center studies are essential to refine candidate selection and mitigate the risks associated with failed attempts in this high-risk obstetric cohort.

## Data Availability

The research data are described in the article presented.
