# Supplementary material for: Successful vaginal birth after two or more cesareans versus failed trial of labor: A systematic review and meta-analysis of maternal and fetal outcomes
Source: Rev Bras Ginecol Obstet. 2026 Sep 23;48:e-rbgo48. doi: 10.61622/rbgo/2026rbgo48 (PMC13690379; doi:10.61622/rbgo/2026rbgo48)
Supplement: Supplementary file 1 [file 1806-9339-rbgo-48-e-rbgo48-Suppl01.pdf]

Chart 1S. Search strategy

Database	Search strategy
PubMed<br>1,506 results	("Vaginal Birth after Cesarean"[Mesh] OR VBAC[tiab] OR CAVB OR "trial of labor after cesarean"[tiab] OR TOLAC[tiab] OR "vaginal delivery after cesarean"[tiab] OR "Cesarean Section, Repeat"[Mesh] OR "two prior cesarean*"[tiab] OR "multiple prior cesarean*"[tiab] OR "repeated cesarean section*"[tiab] OR "previous cesarean section*"[tiab] OR VBAC2[tiab] OR "VBAC-2"[tiab] OR VBAC3[tiab] OR "VBAC-3"[tiab] OR TOLAC2[tiab] OR "TOLAC-2"[tiab] OR TOLAC3[tiab] OR "TOLAC-3"[tiab]) AND (success OR successful OR failed OR failure OR "uterine rupture"[Mesh] OR "uterine rupture"[tiab] OR "maternal morbidity"[Mesh] OR "maternal morbidity"[tiab] OR "fetal morbidity"[Mesh] OR "fetal morbidity"[tiab] OR "neonatal morbidity"[Mesh] OR "neonatal morbidity"[tiab]) NOT (review[pt] OR editorial[pt] OR letter[pt] OR "case reports"[pt])
Embase<br>2,391 results	("Vaginal Birth after Cesarean":ti,ab,kw OR VBAC:ti,ab,kw OR CAVB:ti,ab,kw OR "trial of labor after cesarean":ti,ab,kw OR TOLAC:ti,ab,kw OR "vaginal delivery after cesarean":ti,ab,kw OR "Cesarean Section, Repeat":ti,ab,kw OR "two prior cesarean":ti,ab,kw OR "multiple prior cesarean":ti,ab,kw OR "repeated cesarean section":ti,ab,kw OR "previous cesarean section":ti,ab,kw OR VBAC2:ti,ab,kw OR "VBAC-2":ti,ab,kw OR VBAC3:ti,ab,kw OR "VBAC-3":ti,ab,kw OR TOLAC2:ti,ab,kw OR "TOLAC-2":ti,ab,kw OR TOLAC3:ti,ab,kw OR "TOLAC-3":ti,ab,kw) AND (success:ti,ab,kw OR successful:ti,ab,kw OR failed:ti,ab,kw OR failure:ti,ab,kw OR "uterine rupture":ti,ab,kw OR "uterine rupture":ti,ab,kw OR "maternal morbidity":ti,ab,kw OR "maternal morbidity":ti,ab,kw OR "fetal morbidity":ti,ab,kw OR "fetal morbidity":ti,ab,kw OR "neonatal morbidity":ti,ab,kw OR "neonatal morbidity":ti,ab,kw) NOT (review:ti,ab,kw OR editorial:ti,ab,kw OR letter:ti,ab,kw OR "case reports":ti,ab,kw)
Cochrane<br>166 results	("Vaginal Birth after Cesarean" OR VBAC OR CAVB OR "trial of labor after cesarean" OR TOLAC OR "vaginal delivery after cesarean" OR "Cesarean Section, Repeat" OR "two prior cesarean" OR "multiple prior cesarean" OR "repeated cesarean section" OR "previous cesarean section" OR VBAC2 OR "VBAC-2" OR VBAC3 OR "VBAC-3" OR TOLAC2 OR "TOLAC-2" OR TOLAC3 OR "TOLAC-3") AND (success OR successful OR failed OR failure OR "uterine rupture" OR "uterine rupture" OR "maternal morbidity" OR "maternal morbidity" OR "fetal morbidity" OR "fetal morbidity" OR "neonatal morbidity" OR "neonatal morbidity") NOT (review OR editorial OR letter OR "case reports")

Chart 2S. Outcomes with definitions by the studies

Study	Postpartum hemorrhage (PPH)	Transfusion	Uterine rupture	Neonatal ICU admission
Asakura & Myers, 1995 [12]	Not specified.	Not specified.	1) Rupture: uterine wall defect requiring emergency laparotomy + acute FHR abnormality OR acute maternal bleeding. 2) Dehiscence: uterine wall defect not requiring emergency laparotomy + no fetal compromise or excessive bleeding.	Not specified.
Bretelle et al., 2001 [23]	Implications of bleeding (individual cases), but no numerical definition of PPH.	Not specified.	3 cases of sub-peritoneal scar dehiscence (clinically suspected/confirmed during C-section), but "no frank and complete transperitoneal uterine rupture".	Not specified.
Denjean et al., 2024 [24]	Blood loss > 1000 mL during delivery.	Not specified.	1) Complete: full division of all 3 uterine layers. 2) Incomplete: intact peritoneum, uterine contents within the uterus. Asymptomatic ruptures also reported.	Not specified.
De Leo et al., 2020 [21]	Not specified.	Not specified.	Any symptomatic uterine rupture occurring before delivery.	Not specified.
Metz et al, 2015 [16]	Not specified.	Not specified.	Not specified.	Not specified.
Horgan et al., 2022 [22]	Cumulative blood loss > 1000 mL.	Not specified.	Full-thickness separation of prior uterine scar found at surgery with clinically correlating evidence.	Not specified.
Modzelewski et al., 2019 [15]	Estimated blood loss of 500 mL after vaginal delivery or 1000 mL after C-section (WHO).	Not specified.	Any detected cesarean scar dehiscence, independently of size or clinical symptoms.	Not specified.
Rotem et al., 2021[19]	1) Estimate > 500 mL in VD and > 1000 mL in C-section; OR 2) Blood product transfusion AND/OR Hb drop ≥ 4 g/dL.	Not specified.	Complete uterine scar ruptures. Diagnosis made by attending physician during exploratory laparotomy.	Not specified.
Roux et al., 2023 [18]	Bleeding greater than 500cc, regardless of delivery route.	Not specified.	Rupture of all layers of the uterine wall (including muscle and serosa).	Transfer to neonatal intensive care unit (mentioned).
Spaans et al., 2003 [20]	Not specified.	Not specified.	Not specified.	Not specified.
Maroyi et al., 2023 [17]	Not available.	Not available.	Not available.	Not available.
Gold Zamir et al., 2024 [25]	Early PPH (blood loss exceeding 1000 mL).	Blood product transfusion.	Complete uterine scar rupture (full-thickness defect with direct connection between peritoneal space and uterine cavity), confirmed surgically.	Not specified.
