# Supplementary material for: Successful vaginal birth after two or more cesareans versus failed trial of labor: A systematic review and meta-analysis of maternal and fetal outcomes
Source: Rev Bras Ginecol Obstet. 2026 Sep 23;48:e-rbgo48. doi: 10.61622/rbgo/2026rbgo48 (PMC13690379; doi:10.61622/rbgo/2026rbgo48)
Supplement: Supplementary file 2 [file 1806-9339-rbgo-48-e-rbgo48-Suppl02.pdf]

Supplementary Material

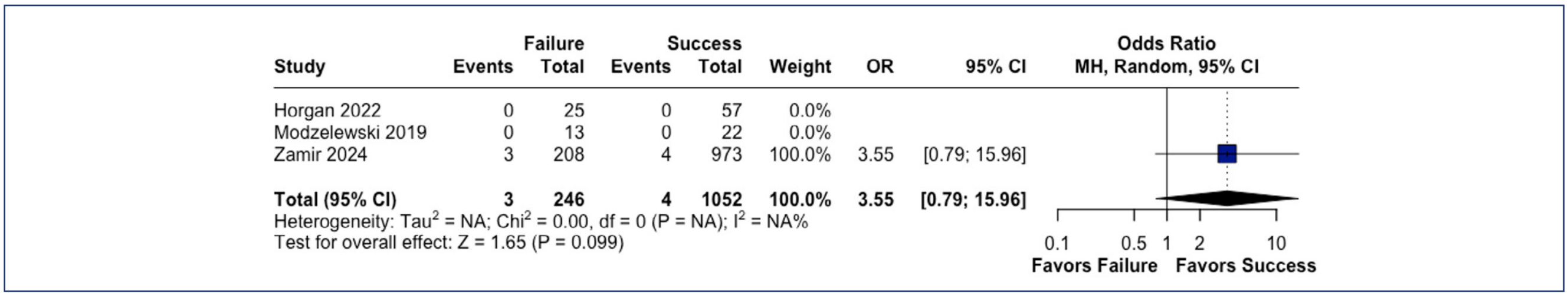

Figure 1S. Pooled results of the outcome of APGAR below 7

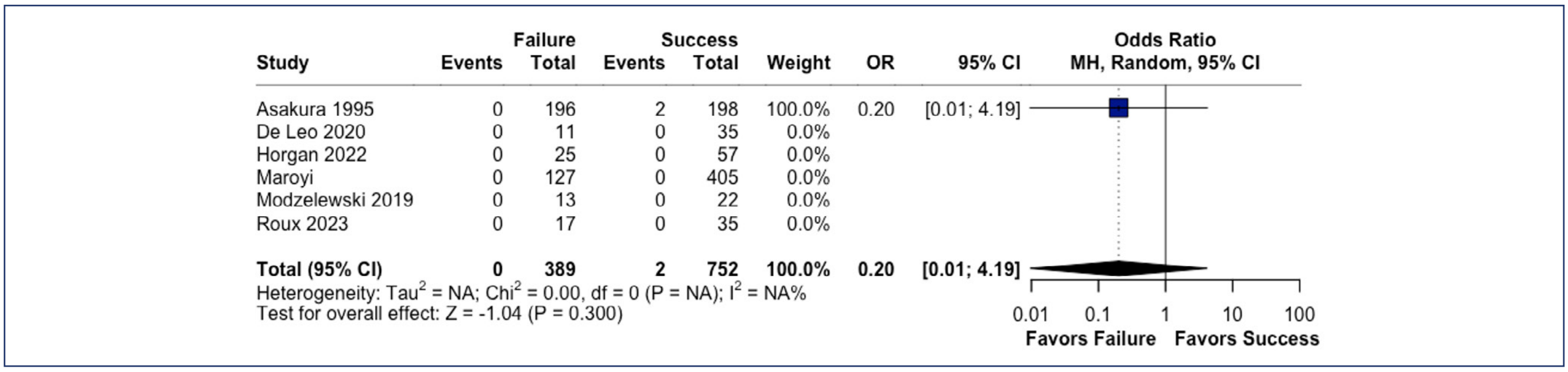

Figure 2S. Pooled results of the outcome of neonatal death

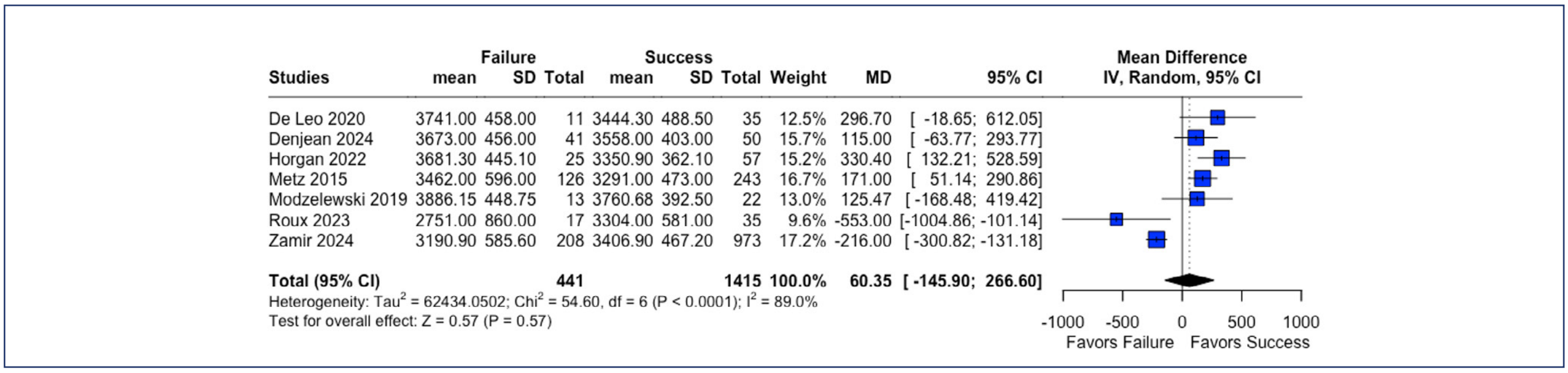

Figure 3S. Pooled results of the risk factor of weight at birth

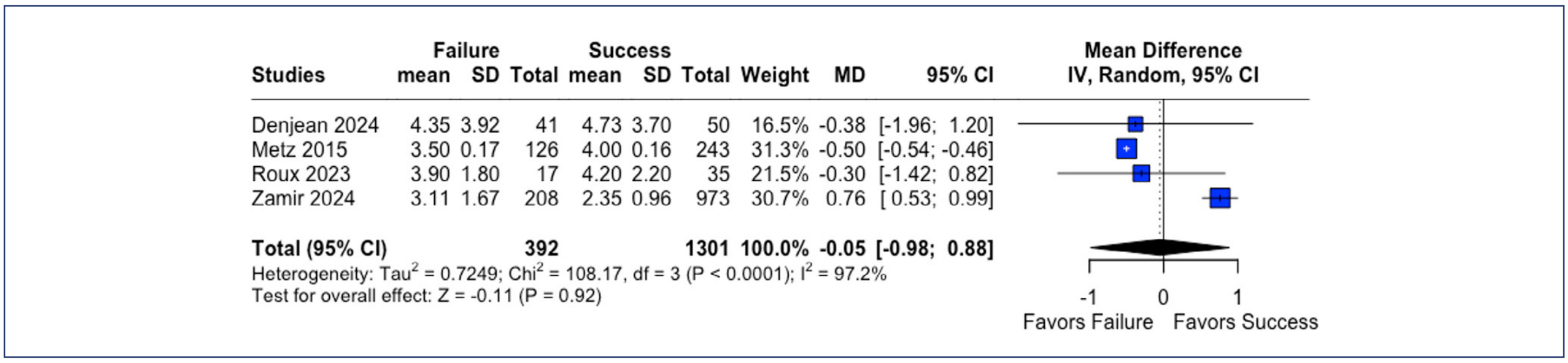

Figure 4S. Pooled results of the risk factor of time since last cesarean section

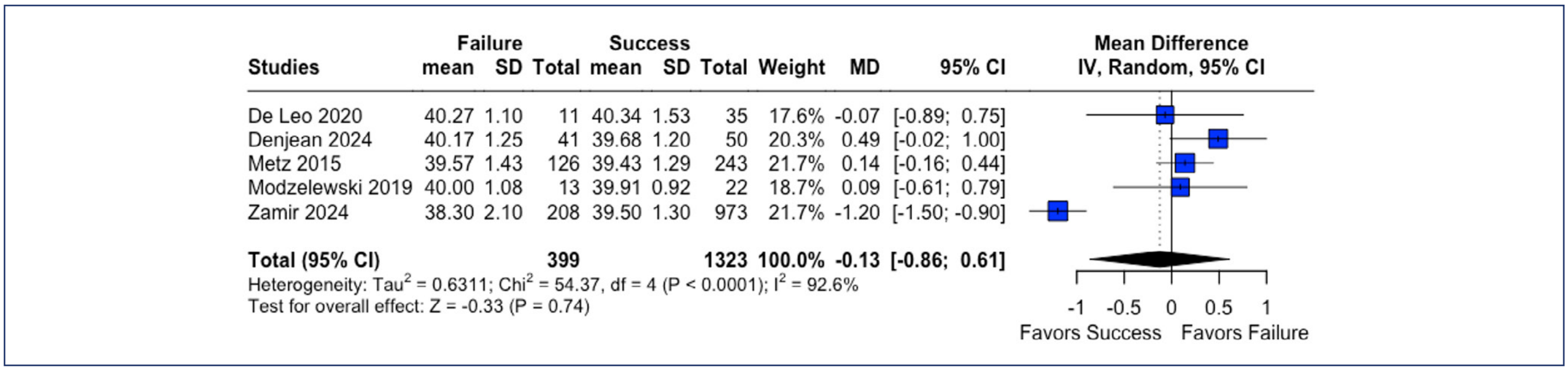

Figure 5S. Pooled results of the risk factor of gestation age at delivery

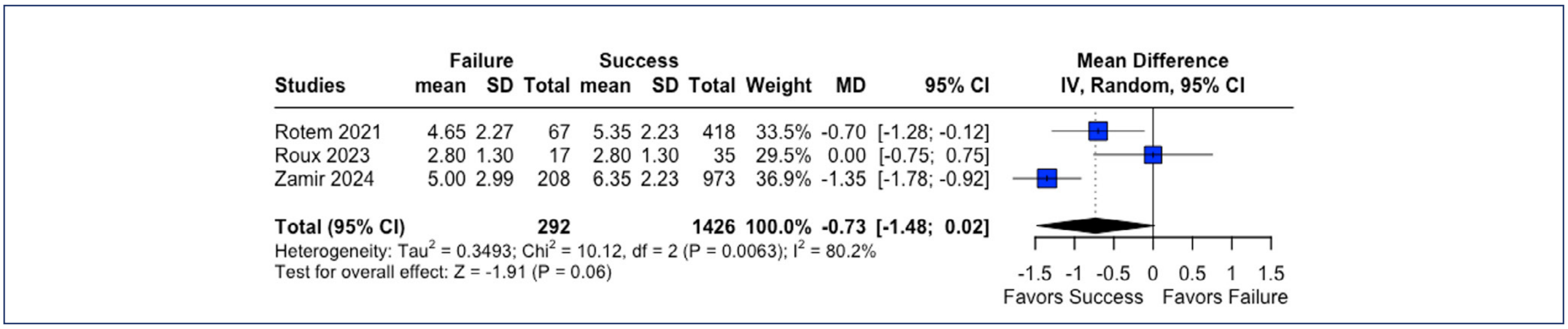

Figure 6S. Pooled results of the risk factor of mean parity

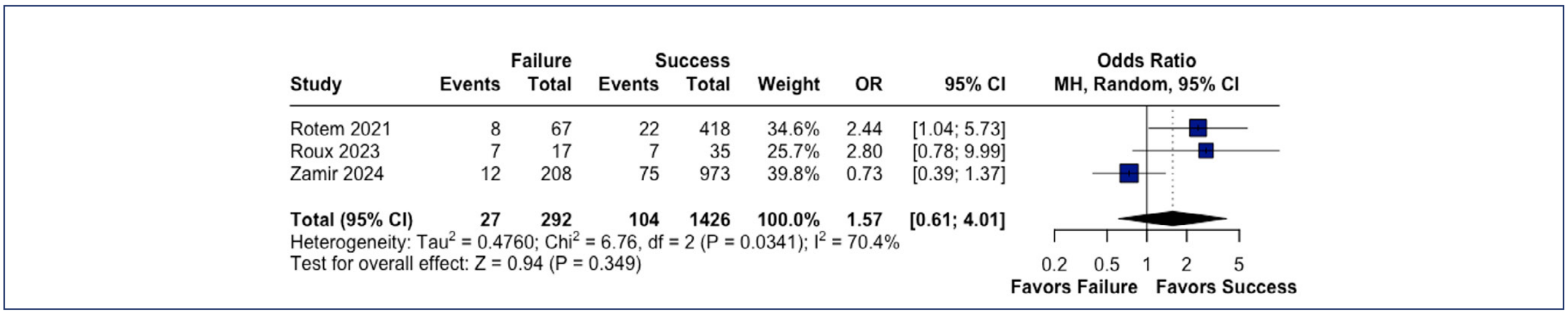

Figure 7S. Pooled results of the risk factor of diabetes (Type I, Type II or Gestational diabetes)

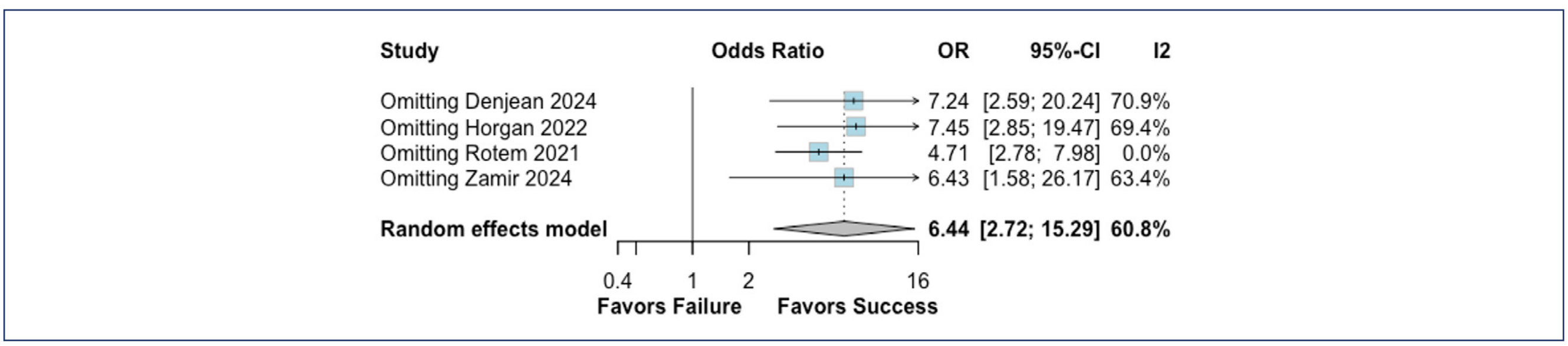

Figure 8S. Leave-one-out analysis of the outcome of neonatal ICU admission

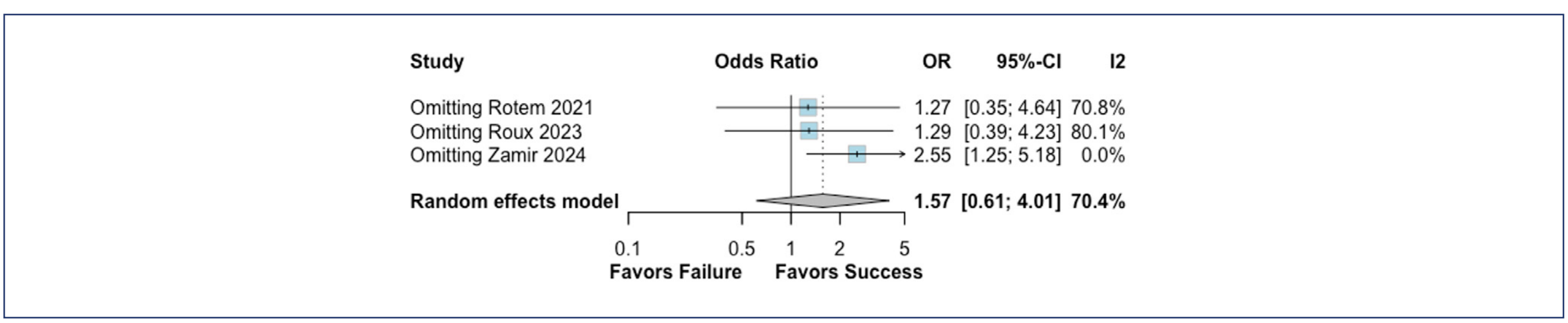

Figure 9S. Leave-one-out analysis of the risk factor of diabetes

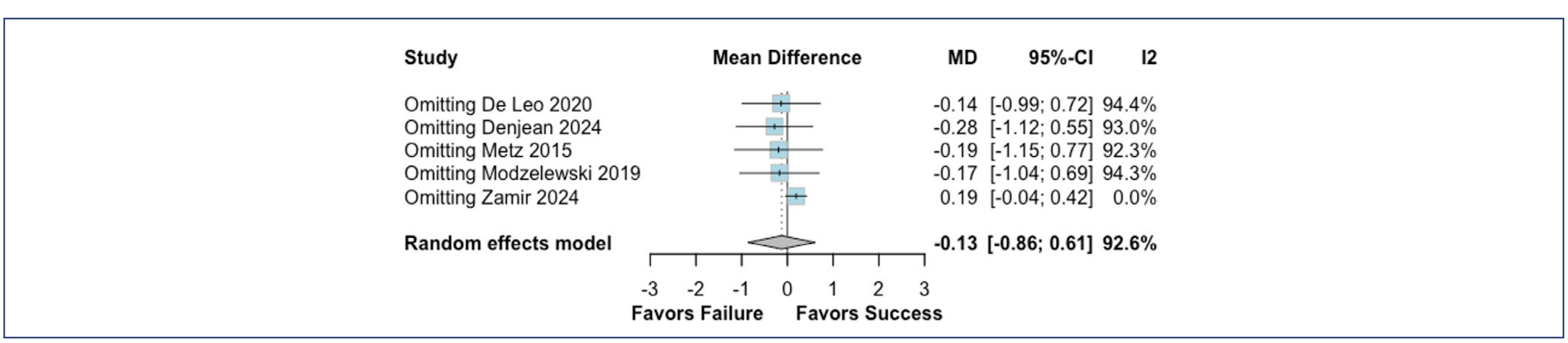

Figure 10S. Leave-one-out analysis of the risk factor of gestational age

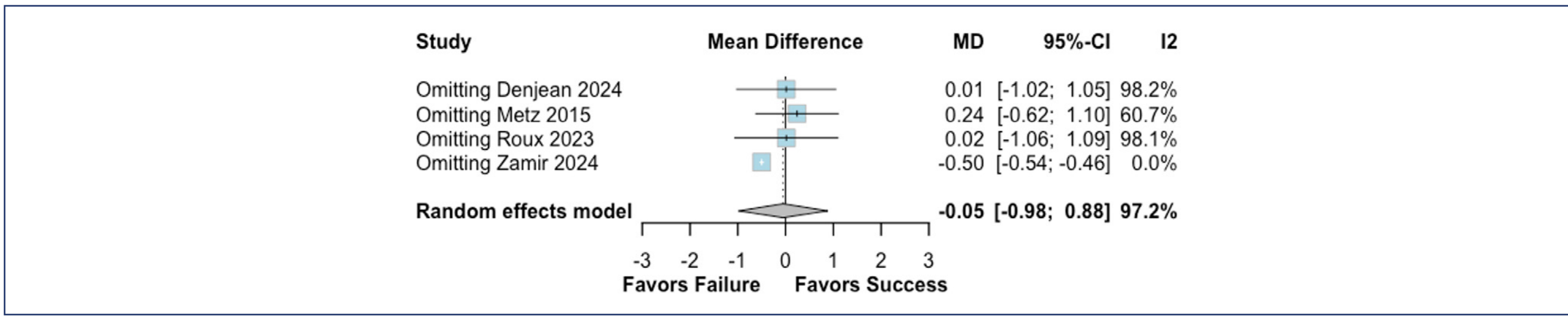

Figure 11S. Leave-one-out analysis of the risk factor of time since the last cesarean section

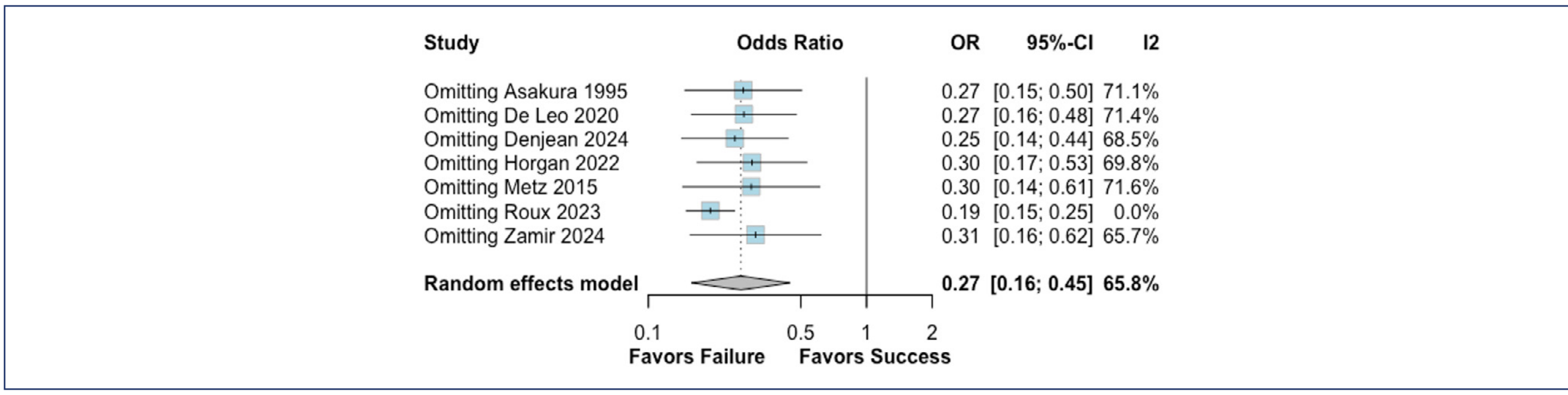

Figure 12S. Leave-one-out analysis of the risk factor of history of vaginal delivery

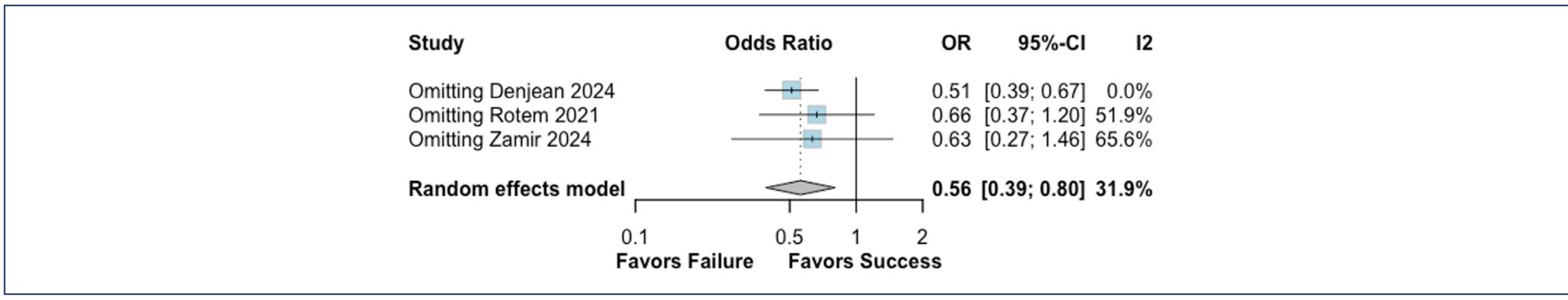

Figure 13S. Leave-one-out analysis of the risk factor of peridural anesthesia

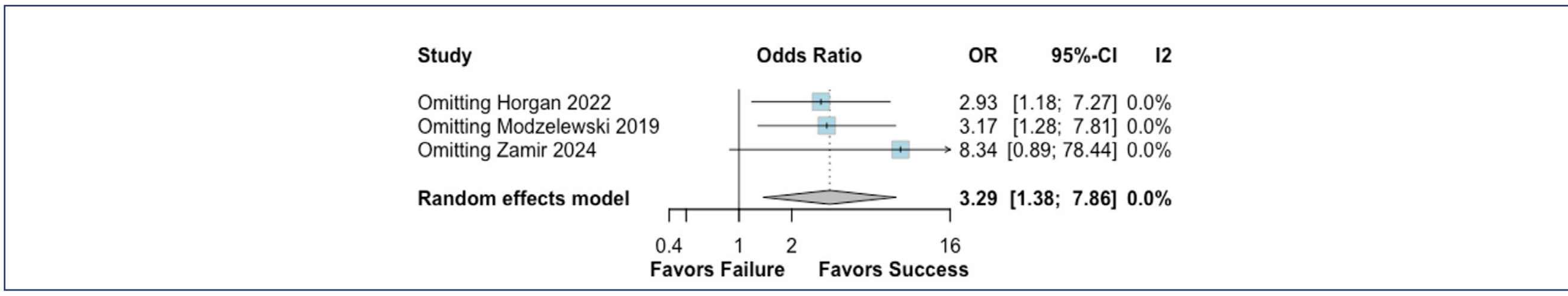

Figure 14S. Leave-one-out analysis of the outcome of transfusion

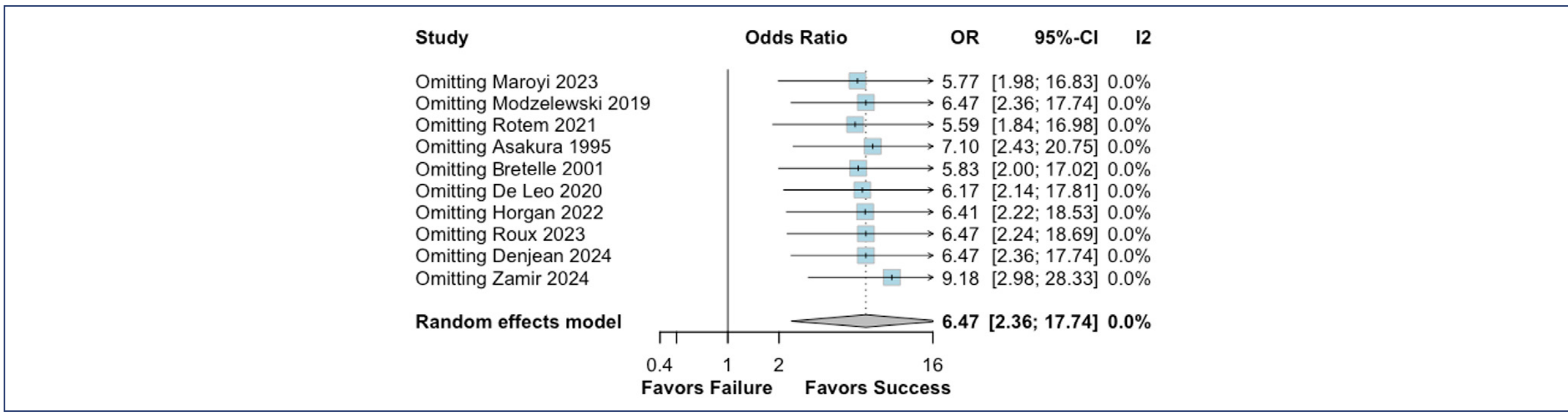

Figure 15S. Leave-one-out analysis of the outcome of uterine rupture

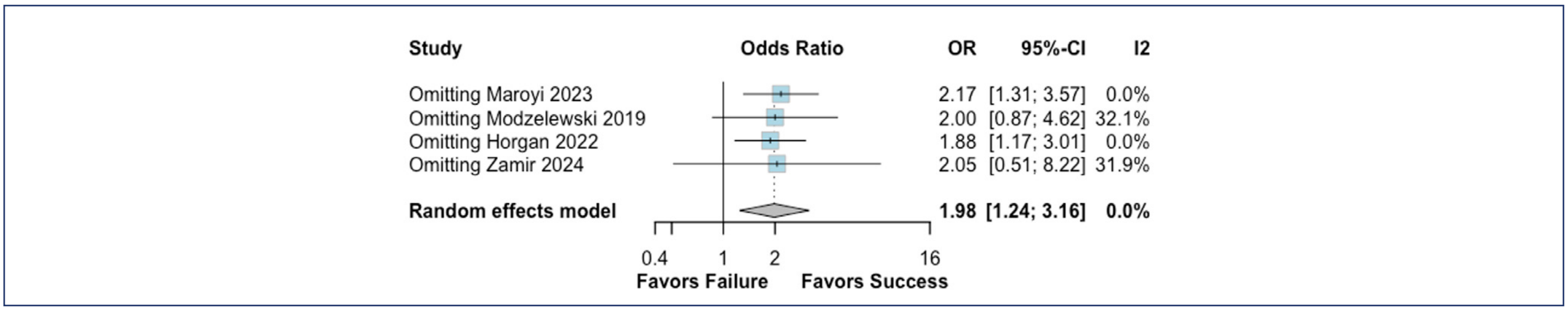

Figure 16S. Leave-one-out analysis of the outcome of hemorrhage

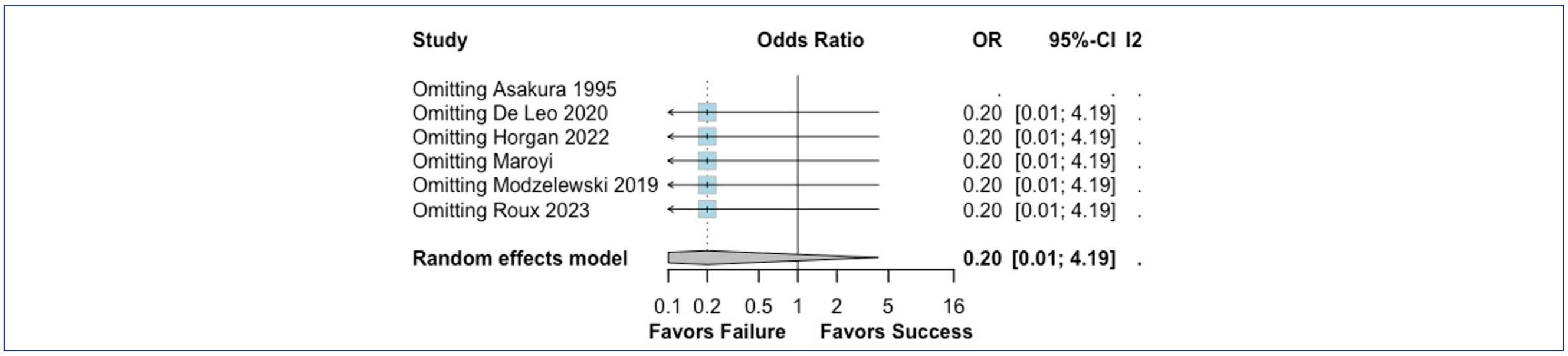

Figure 17S. Leave-one-out analysis of the outcome of neonatal death

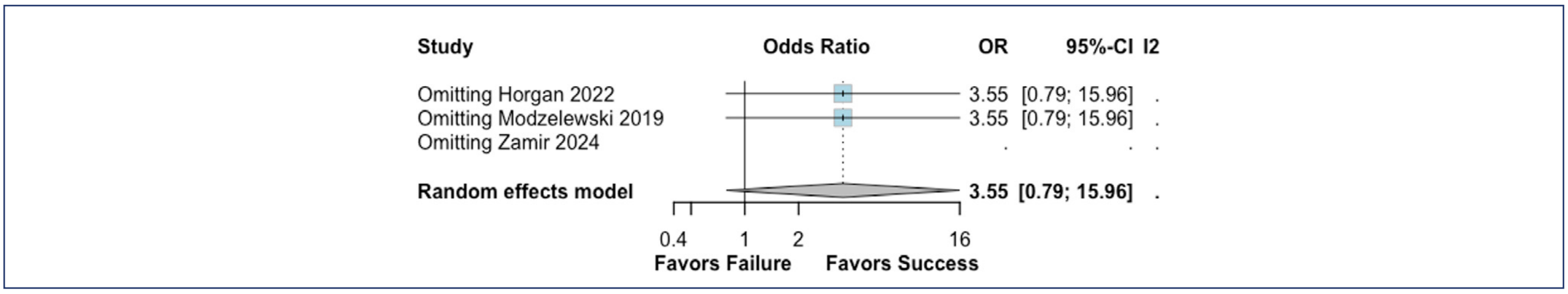

Figure 18S. Leave-one-out analysis of the outcome of APGAR below 7

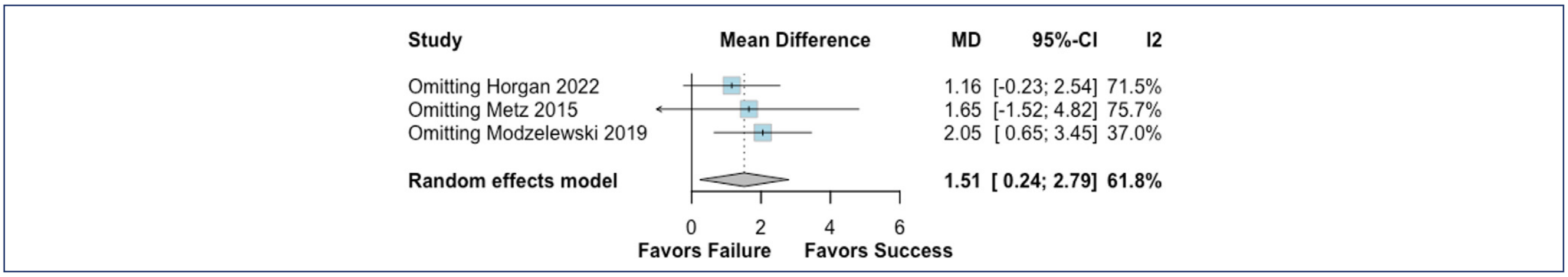

Figure 19S. Leave-one-out analysis of the risk factor of BMI

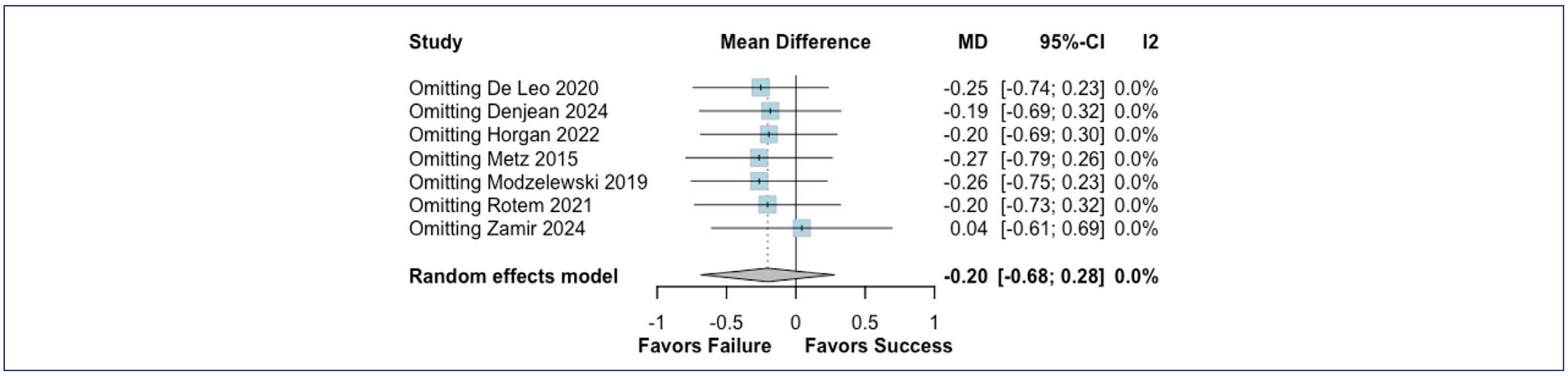

Figure 20S. Leave-one-out analysis of the risk factor of age

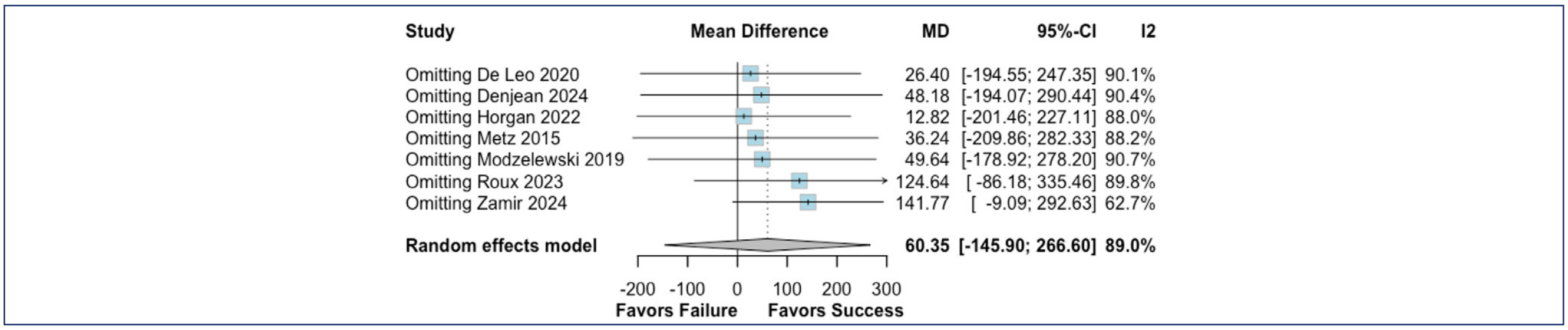

Figure 21S. Leave-one-out analysis of the risk factor of weight at birth

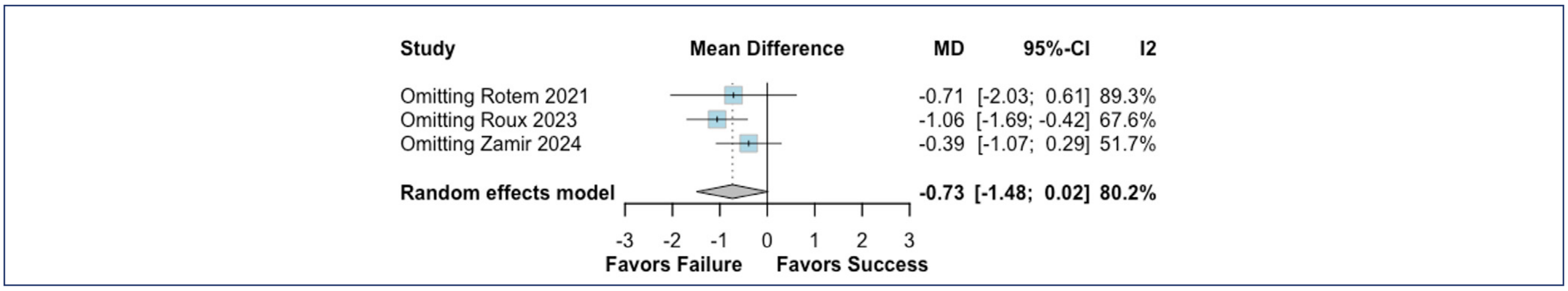

Figure 22S. Leave-one-out analysis of the risk factor of parity
